# Health Assessments of Common Bottlenose Dolphins (*Tursiops truncatus*): Past, Present, and Potential Conservation Applications

**DOI:** 10.3389/fvets.2019.00444

**Published:** 2019-12-13

**Authors:** Ashley Barratclough, Randall S. Wells, Lori H. Schwacke, Teresa K. Rowles, Forrest M. Gomez, Deborah A. Fauquier, Jay C. Sweeney, Forrest I. Townsend, Larry J. Hansen, Eric S. Zolman, Brian C. Balmer, Cynthia R. Smith

**Affiliations:** ^1^National Marine Mammal Foundation, San Diego, CA, United States; ^2^Chicago Zoological Society's Sarasota Dolphin Research Program, Mote Marine Laboratory, Sarasota, FL, United States; ^3^NOAA, National Marine Fisheries Service, Office of Protected Resources, Silver Spring, MD, United States; ^4^Dolphin Quest, San Diego, CA, United States; ^5^Bayside Hospital for Animals, Fort Walton Beach, FL, United States

**Keywords:** dolphin, *Tursiops truncatus*, conservation, health assessment, veterinary medicine

## Abstract

The common bottlenose dolphin (*Tursiops truncatus*) is a global marine mammal species for which some populations, due to their coastal accessibility, have been monitored diligently by scientists for decades. Health assessment examinations have developed a comprehensive knowledge base of dolphin biology, population structure, and environmental or anthropogenic stressors affecting their dynamics. Bottlenose dolphin health assessments initially started as stock assessments prior to acquisition. Over the last four decades, health assessments have evolved into essential conservation management tools of free-ranging dolphin populations. Baseline data enable comparison of stressors between geographic locations and associated changes in individual and population health status. In addition, long-term monitoring provides opportunities for insights into population shifts over time, with retrospective application of novel diagnostic tests on archived samples. Expanding scientific knowledge enables effective long-term conservation management strategies by facilitating informed decision making and improving social understanding of the anthropogenic effects. The ability to use bottlenose dolphins as a model for studying marine mammal health has been pivotal in our understanding of anthropogenic effects on multiple marine mammal species. Future studies aim to build on current knowledge to influence management decisions and species conservation. This paper reviews the historical approaches to dolphin health assessments, present day achievements, and development of future conservation goals.

## Introduction

Assessment of marine mammal health is complex, both from an accessibility standpoint and from the diverse array of factors influencing both individual and population survival. Baseline data are particularly crucial to evaluate whether population, or in some cases species, health is deteriorating by providing points of comparison to assess trends in disease, mortality, and reproductive rates (1). From a veterinary perspective, a hands-on physical exam is the optimal approach to provide critical information for a comprehensive understanding of both individual and population-level health status. Knowledge gained from physically examining smaller cetaceans can be extrapolated to larger, less-accessible cetaceans to improve understanding of the complexities of adverse health impacts on marine mammal conservation. The common bottlenose dolphin (*Tursiops truncatus*) is an effective model species for understanding both cetacean and marine ecosystem health.

The overarching aim of conservation is to actively preserve habitats and the diversity of species dwelling therein (2). Effective conservation benefits from a foundation of sound scientific understanding of species' biology, population dynamics, and stressors impacting the ecosystem (3). Marine ecosystem health is particularly challenging to assess due to immense biological diversity, as well as the vast scale and connectivity of the ocean environment. Multiple factors threaten marine mammal health (4); therefore, improving knowledge of the interplay of these factors and predicting their long-term effects are essential for successful conservation.

The emerging discipline of conservation physiology is particularly important in the marine environment, as it allows a mechanistic understanding of the drivers of conservation obstacles at an ecosystem level, in addition to species-specific challenges. Understanding how species physiologically respond to environmental alterations is important for successful tailored conservation strategies (5). The rate of expansion of the human population is exacerbating the challenges faced by wildlife with frequently detrimental consequences to their health (6). The unprecedented changes occurring in the environment on a global scale require novel mitigation strategies to ensure effective conservation actions and sustainable wildlife populations (7).

There is limited understanding of the scale of the negative anthropogenic impact on marine mammal biodiversity. Currently 29.2% of marine mammal species are classified as data deficient according to the International Union for the Conservation of Nature (8). As a result, shifts in population viability can be difficult to detect, as the basic natural history of the species has not been documented (9). Intrinsic traits of species can be more important predictors of risk than extrinsic environmental factors, as they provide a measure of the species' inherent susceptibility to human impacts and the ability of species to recover from them (10). The bottlenose dolphin is one of the most closely studied and widely distributed marine mammal species and provides an opportunity to extrapolate knowledge and understanding to other marine mammal species.

## Historical Perspective

**Figure 1:**
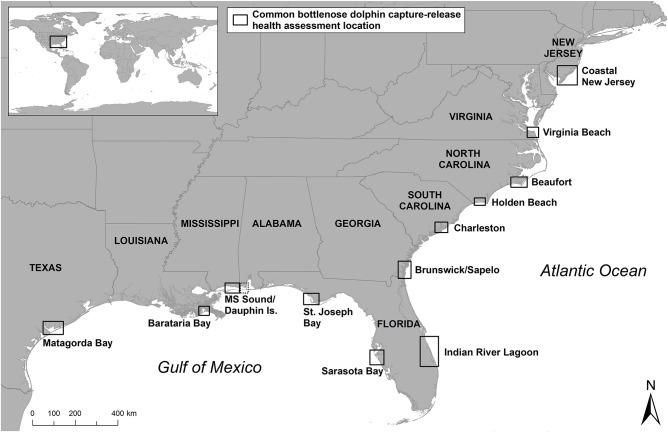
Bottlenose dolphin capture-release health assessment locations in the U.S.

Initially the first common bottlenose dolphin captures occurred for acquisition for public display, research, or for stock assessment prior to collection. Over time health assessments of inshore dolphins have been utilized to better understand endemic disease, establish baseline physiological measures, and evaluate exposure to, and potential effects of, chemical, biological, and physical stressors. Historically, health assessments developed as additions to existing capture-release efforts for other purposes, such as marking/tagging and population-level studies to understand movement patterns and site fidelity. Starting in 1979, Hubbs/SeaWorld Research Institute began collecting bottlenose dolphin health data in the Indian River Lagoon of east Florida during capture-release operations to assess potential population-level impacts in advance of upcoming zoologic collections (Figure 1) (11). Analyses from the samples and data collected included morphometrics, blood biochemistry, hematology and reproductive endocrinology, microbiology, genetics, and life history studies (12). Similar research was initiated by Marine Animal Productions in 1982 in Mississippi Sound, with the inclusion of skin, blubber, and liver tissue sampling in some cases, to establish a health baseline for dolphins inhabiting this area, again prior to collections and for comparison with managed animals (13).

The Sarasota Dolphin Research Program (SDRP) began incorporating additional biological samples and measurements in 1984 to their original capture-release program. This supported tagging and telemetry studies in Sarasota Bay, Florida, which were initiated in 1970. The inclusion of additional biological samples shifted the focus from studies of population range and social patterns to broader scientific investigations of life history, population dynamics, body condition and health, social structure, communication, reproductive success, and effects of human interactions. Samples and measurements included in-depth bloodwork analyses, genetic tests for population structure and paternity, ultrasonic measurement of blubber thickness, weight measurement, age determination (tooth growth layer group counts), further development of tags and tag attachments, and post-release population monitoring (14–19).

In 1992, the Marine Mammal Health and Stranding Response Program was formalized within the National Marine Fisheries Service (NMFS) through an amendment to the Marine Mammal Protection Act (MMPA), establishing the standards for sample collections and promoting collaboration and standardization of bottlenose dolphin health assessments (20). In 1995, NMFS conducted health assessments in response to the 1987–88 mortality event along the east coast of the U.S., in which over 600 bottlenose dolphins stranded as a result of a large-scale morbillivirus epizootic (21–25). Additional studies were also conducted to investigate unusual increases in dolphin strandings near Matagorda Bay, Texas in 1992 (26), and in the Florida Panhandle in 2005–2006 (27) (Figure 1) (Table 1).

**Table 1:** Historical list of previous capture locations according to Figure 1 including number of animals examined or health assessments (HA) performed, purpose of captures, and references.

**Date**	**Location**	**Number of dolphins**	**Purpose**	**References**
1979–1981	Indian River Lagoon FL	27 109 total HA	Population assessment	(11)
1982	Mississippi Sound MS	57	Commercial assessment	(13, 28)
1984–2019 (ongoing)	Sarasota FL	289 individuals 811 total HA	Biological sampling, technique development, reference population	(29)
1987	Virginia Beach VA	23	Mass mortality investigation	(21)
1992	Matagorda Bay TX	36	Mortality investigation	(26)
1995	Beaufort NC	31	HA post CeMV outbreak	(24)
1998	Virginia Beach VA	1	Stock assessment	NOAA unpublished data
1999	Charleston SC	14	Stock assessment	(30)
1999^*^ 2000^*^ 2006^*^	Beaufort NC	6 11 19	Stock assessment	(31) (30) (32)
2002–2003	Brigantine NJ	12	Stock assessment	(30)
2004	Holden Beach NC	10	Persistent organic pollutant assessment	(33)
2003–2018	Charleston SC Indian River Lagoon FL	118 246	Comparative health studies	(34)
2005–2006	St. Joseph Bay FL	30	UME investigation	(27)
2009	Brunswick GA	29	HA legacy environmental contamination	(35)
2011–2018	Barataria Bay LA	202	DWH investigation	(36)
2013	Mississippi Sound MS	20	DWH investigation	(37)
2015	Brunswick GA	19	UME investigation	(38)
2018	Dauphin Island AL	18	DWH investigation	

* *The numbers provided are for those where samples were used and published it does not necessarily provide a list of the total numbers of animals handled in this location*.

Supplementary capture-release studies were initiated by the National Oceanic and Atmospheric Administration (NOAA) in support of stock assessment on the east coast, which was by that time a requirement for NOAA's NMFS (23, 24). Subsequent capture-release studies used telemetry to understand population structure along the Atlantic coast (39) following increased dolphin mortality (40) which was eventually determined to be associated with morbillivirus (22, 30). The studies found positive morbillivirus titers in some dolphins sampled in estuarine and coastal waters near Beaufort, NC, with no positive titers observed in dolphins sampled in estuaries near Charleston, SC (30). This provided crucial information to understanding dolphin population structure and interaction along the U.S. east coast, with a mosaic of migratory, non-migratory but coastal, and small estuarine stocks rather than a single large population as initially presumed. In addition utilizing archived Sarasota Bay samples to retrospectively assess morbillivirus titers facilitated further understanding of virus exposure, seroconversion, and population naivety (41). Incorporating health assessments informed scientists that at least some small, estuarine stocks along the Atlantic coast were naïve to morbillivirus and therefore particularly vulnerable (38).

In 2003, the Health and Environmental Risk Assessment (HERA) population monitoring project was initiated for bottlenose dolphins in the Indian River Lagoon, Florida (IRL), and waters surrounding Charleston, South Carolina (CHS) (42). For both the IRL and CHS field sites, health assessments were conducted to establish baseline data and to compare morbidity temporally and across two geographic sites (34, 43). Higher concentrations of persistent organic pollutants (POPs) including legacy [e.g., dichlorodiphenyltrichloroethanes (DDTs), polychlorinated biphenyls (PCBs)] as well as “emerging” contaminants [polybrominated diphenyl ethers (PBDEs) and perfluorooctane sulfonate (PFOS) compounds] were detected in CHS dolphins as compared to IRL dolphins (43, 44). Mercury concentrations in the blood and skin of IRL dolphins were extremely high, approximately five times higher than those in CHS dolphins. Higher exposure to many pathogens (e.g., morbillivirus and lobomycosis) was also observed for the IRL dolphins (34).

The incorporation of health assessments into existing capture-release protocols provided the collection of baseline health data across multiple populations, thus allowing for investigation of geographic variability in health parameters (32, 42). The methodologies established and samples collected during these earlier projects formed the basis for developing a risk assessment framework to quantify the impacts for dolphins affected by anthropogenic threats (e.g., *Deepwater Horizon* oil spill) in geographical areas where baseline data were not available (36).

## Threat Identification and Assessment

Dolphin capture-release projects provide a unique perspective to assess individual animal health and extrapolate to overall health of the surrounding population, species, and ecosystem. Over the past 40 years in the U.S., there have been numerous stressors that have impacted bottlenose dolphin populations, for which dolphin capture-release projects have been integral to threat identification and quantification of impacts from a given stressor (e.g., biotoxins, disease, environmental contaminants, oil spills, etc.).

### Unusual Mortality Events

An unusual mortality event (UME) is defined under the U.S. Marine Mammal Protection Act as “a stranding that is unexpected; involves a significant die-off of any marine mammal population; and demands immediate response.” The UME program was officially established under Title IV of the MMPA in 1992. Increased recognition of the occurrence of large scale dolphin mortality events in the late 1980s spurred the application of dolphin health assessments beyond population monitoring, to investigating causes and effects of such mortality events (21, 24).

Since 1999, a series of UMEs occurred along the northwestern Florida coastline (Florida Panhandle) including St. Joseph Bay (45). NOAA conducted two dolphin health assessments during 2005 and 2006 in response to these UMEs in which 30 dolphins were sampled and subsequently tagged (27) (Table 1). The initial mortalities were tentatively attributed to biotoxins from red tide algae (*Karenia brevis*) (46). Eosinophilia was observed in 23% of sampled dolphins, with associated increased neutrophil phagocytosis and T-lymphocyte proliferation (27). Chronic low-level exposure to another algal toxin, domoic acid, produced by the diatom *Pseudo-nitzschia* spp., previously linked to eosinophilia, was also identified (47). Prior to these UMEs, little was known about dolphin abundance, distribution, and site fidelity in this region, and thus, it was unclear which population(s) of dolphins were impacted (48, 49). Post-health assessment tagging data suggested that the timing and spatial extent of biotoxin events and other potential stressors in the Florida Panhandle may greatly influence the severity of future UMEs (50). Cetacean post-mortem examinations can provide insight into identifying the underlying cause of a UME in addition to baseline data on disease presence and anthropogenic causes of mortality (51–53). Increased integration of live animal assessment with post-mortem findings encourages the transfer of information from the dead to the living, informing scientists of the underlying pathophysiological mechanisms faced within free-ranging populations (54).

### Chemical Pollutants

The presence of PCBs and other lipophilic contaminants have been recorded to be accumulating in the tissues of bottlenose dolphins and other odontocetes for decades (31, 55–59). Many marine mammals, particularly piscivorous species, have a high potential to biomagnify pollutants (33), with increased levels resulting from the high trophic position and blubber acting as a reservoir for lipophilic contaminants (60). Both experimental and observational studies support the correlation between increased PCB levels and endocrine dysfunction, compromised immunity, and/or reproductive failure (61–66). However, the common co-occurrence of similarly acting compounds and uncertainty regarding species-specific dose response functions makes assessment of the effects of these contaminants at a population level challenging (60, 67).

In addition to their applicability to investigating UMEs, dolphin health assessments have also been used to investigate health of populations at risk from environmental contaminants. For example, dolphins along the Georgia coast have been identified with some of the highest concentrations of PCBs in the world, and these levels are site-specific to a Superfund Site in Brunswick, Georgia (60, 68, 69). In 2009, NOAA conducted health assessments on 29 dolphins in the region and identified a high proportion (26%) of sampled individuals suffered from anemia (66). In addition, these dolphins had reduced thyroid hormone levels with total thyroxine, free thyroxine, and triiodothyronine negatively correlated with increased blubber PCB concentrations. T-lymphocyte proliferation and indices of innate immunity decreased with blubber PCB concentration, suggesting an increased susceptibility to infectious disease (66). As with previously described health assessments, telemetry and photo-ID data provided perspective on ranging patterns relative to exposure, subsequent reproductive success, and effects of long-term impacts from cumulative stressors (70). In 2015, another health assessment was conducted in Georgia to look at potential impacts associated with a recent morbillivirus-caused UME and extremely high levels of PCBs that were identified from previous studies. Dolphin morbillivirus titers differed between dolphins sampled in coastal and estuarine waters, and tagging data identified some degree of overlap between these individuals. This study suggested that estuarine dolphins in this region may be highly susceptible to future morbillivirus infections as a result of elevated PCB levels and spatial overlap between coastal and estuarine dolphins that would facilitate disease transmission (50).

### Petroleum Toxicity

In 2010, the largest marine oil spill in the history of the U.S., the *Deepwater Horizon* oil spill (DWH), occurred in the northern Gulf of Mexico (GoM). Subsequently a multidisciplinary approach for evaluating the impacts upon cetaceans was undertaken (71, 72). Bottlenose dolphins were the focal cetacean species examined due to the accessibility of dolphins in shallow coastal and estuarine waters, and the heavy oiling in some of those same nearshore areas. Health assessments in heavily oiled areas, particularly Barataria Bay, Louisiana, were initiated as part of NOAA's Natural Resource Damage Assessment (NRDA). Veterinary clinical assessment of dolphins living within the oil spill footprint found significant lung pathology, impaired stress responses, high reproductive failure, and altered functional immunity, as compared to findings from un-oiled reference populations (35, 73–75). After the DWH oil spill, an abnormally high prevalence of lung and adrenal gland pathologies were documented in post-mortem examinations (73, 76). Identifying the baseline incidence of disease in stranded dolphins and diagnosing pathology in live dolphins through the application of enhanced health assessment protocols was required to determine if these findings were correlated with the recent environmental exposure (75, 77, 78).

Determining a causal link for the multiple pathologies observed post DWH oil exposure has been via a diagnosis of exclusion; concluding the toxic effects of the oil spill as the primary differential to both the observed pathologies and the increased dolphin mortality (71, 75). Other differential diagnoses that were the potential causes of previous GoM UMEs were also ruled out including biotoxins (79), POPs (68), and infectious disease (45, 73, 80, 81). Successive health assessments during 2016–2018 have provided additional insight into the chronic health effects. Longitudinal photo-ID surveys allowed estimation of post-spill survival rate (0.80–0.85) and reproductive success (20%), the latter was very low for this population (82, 83). Long-term consequences from oil contamination are difficult to assess but have been suggested in other marine mammals such as the killer whale (*Orcinus orca*) and the sea otter (*Enhydra lutris nereis*) (84–87). Future research efforts will aim to improve understanding of the transgenerational or *in utero* exposure effects in addition to the direct exposure of those animals alive at the time of the oil spill.

Currently there is limited knowledge of the pathophysiology of reproductive failure in bottlenose dolphins. Improved understanding of the normal physiological changes occurring during gestation in successful pregnancies will aim to elucidate possible mechanisms of reproductive failure and identify abnormalities occurring during failed pregnancies. This knowledge will be essential to help understand reproductive challenges, not only for common bottlenose dolphins, but also for the future management of other cetaceans, some critically endangered, to help to understand the reproductive challenges faced in these species and improve future conservation management.

## Capture and Handling Methodology

**Figure 2:**
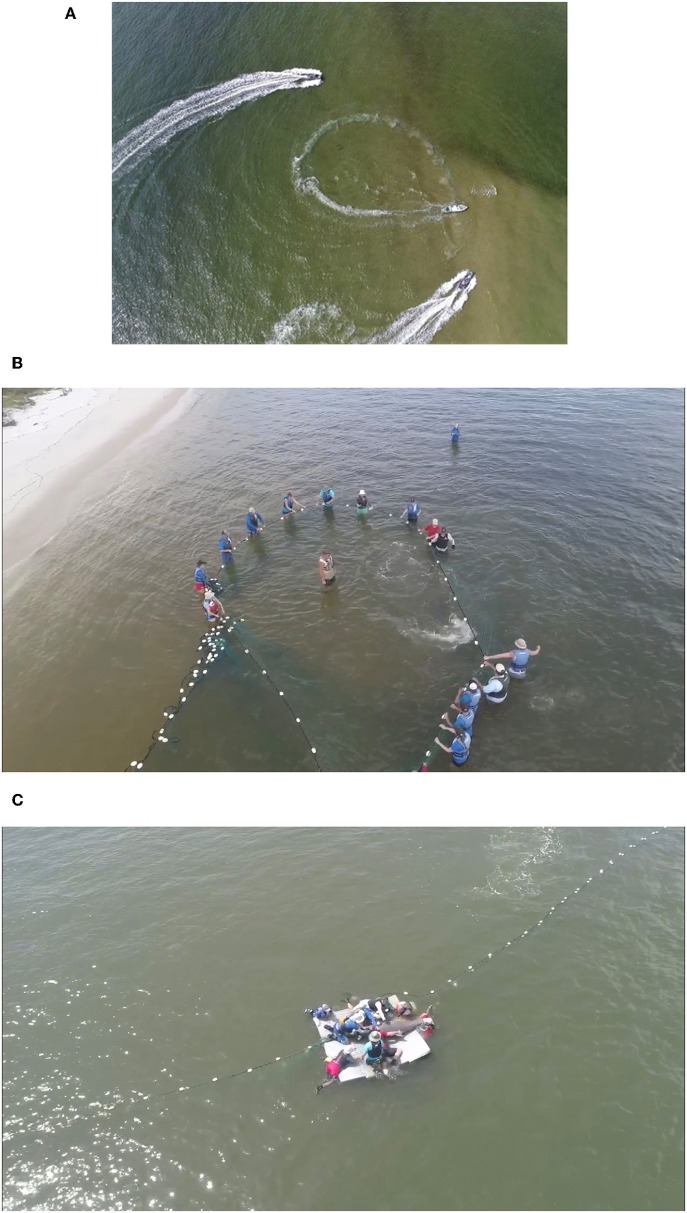
Capture methodology with **(A)** seine net deployed from a specially designed boat creating a compass in the center of the image, with chase boats circling outside to help contain the animals before completion of the compass and to deliver handlers to the net (two dolphins are visible inside the compass on the left side). **(B)** Shallow water set, well-trained handlers distributed around the circumference of the compass to provide aid and restraint when the dolphins contact the net. **(C)** Deep water set, dolphin is placed onto a floating mat and disentangled from the net for transport to the processing vessel. All photos taken under NMFS MMPA/EAS permit No. 18786-03.

The standard approach to capture small (1–5) numbers of bottlenose dolphins in shallow waters is by encirclement with a seine net up to 500 m long and 7 m deep (88, 89). Shallow water (<1.5 m), minimal currents, and a solid seafloor are optimum for safe capture and restraint. The seine net is deployed from a specially designed boat at high speed around the target dolphin(s), creating a compass (Figure 2A), with well-trained handlers distributed around the circumference to provide aid and restraint when the dolphins contact the net (Figure 2B) (29). If capture occurs in deep water (>1.5 m), the net compass can be pulled into nearby shallow water, or the dolphins are handled from the side of response vessels and moved onto specially designed floating mats that are either towed to shallow water or directly onto a processing vessel for sample collection (Figure 2C). Capture of individual dolphins in waters exceeding the depth of seine nets has been performed via tail grabs or hoop-nets, which are placed over the head of bow-riding bottlenose dolphins or smaller cetacean species as they surface to breathe (88–90). Once dolphins are safely restrained, veterinary examinations and sampling are performed.

## Veterinary Processing

Current health assessment examinations require collaboration among scientists from multiple disciplines and veterinary specialties to obtain the maximum amount of information from a single snapshot in time while the animal is in-hand. The development of veterinary field techniques was enhanced by the successful management of dolphins in human care. Sampling varies according to research questions, but typically a suite of baseline data are collected to maximize knowledge obtained from a single veterinary exam (Table 2) (91). Ensuring a standardized approach to highlight the importance of inter-lab comparability and sharing information between multiple institutions has been paramount in the success of developing field collection methodologies and subsequent sample analysis procedures (32, 113). As described below, additional field procedures have been incorporated over the decades as technology, field techniques, and analytical assays have advanced, and as management needs have evolved.

**Table 2:** List of veterinary processing sample collection from hands-on physical examinations during common bottlenose dolphin health assessments.

**Procedure**	**Description**	**Use**	**References**
Blood sample (Figure 4)	Obtained from the periarterial rete on the ventral aspect of the tail fluke	Biochemistry Hematology Blood gas analysis Endocrinology Immunology Serology Genetics	(23, 29, 30, 74, 91–93)
Surgical biopsy	Full thickness wedge biopsies of skin and blubber are routinely taken via an inverted “L” block under local anesthesia from the left lateral body wall caudal to the dorsal fin	Genetic population structure (skin) Foraging ecology (skin) Chemical contaminants (blubber) Hormone levels (blubber) Microbiome	(31, 33, 49, 57, 94–96)
Urinalysis	Bladder catheterization	Renal function assessment Dietary analysis	(91, 97–99)
Tooth extraction	Single tooth extracted under local anesthesia	Age determination	(17, 100)
Ultrasonography (Figure 3)	Thoracic and abdominal internal assessment	Lung pathology Reproductive Assessment Full abdominal exam including renal assessment Blubber thickness	(77, 97, 101–103)
Electrocardiography (Figure 6)	Adapted field use in and out of water	Cardiac assessment	(104)
Morphometrics (Figure 7)	Standardized full body measurements: lengths, girths, weight	Assess body condition and growth rates	(105)
Auditory evoked potential	Portable unit adapted for field assessment audiograms	Assess hearing range and sensitivity	(106)
Lesion biopsy	Sample of abnormal skin lesions e.g., pox or freshwater lesions	Histopathology	(57, 93)
Blow analysis (Figure 5)	Exhaled breath vapor	Pathogen and hormonal analysis Metabolites Respiratory function testing	(107, 108)
Microbiology	Swabs/culture plates from oral respiratory or genital orifices	Bacteriology Virology	(109)
Freeze brand	Dorsal Fin	Identification	(110)
Feces and urine collection	Swabs or catheter	Biotoxin analysis	(46)
Skin biopsy Electronic and/or roto tagging	Skin sample from biopsy or during dorsal fin tagging	Genetics, sex, stable isotopes identification Ranging patterns reproductive status Survival	(70, 111, 112)

**Figure 3:**
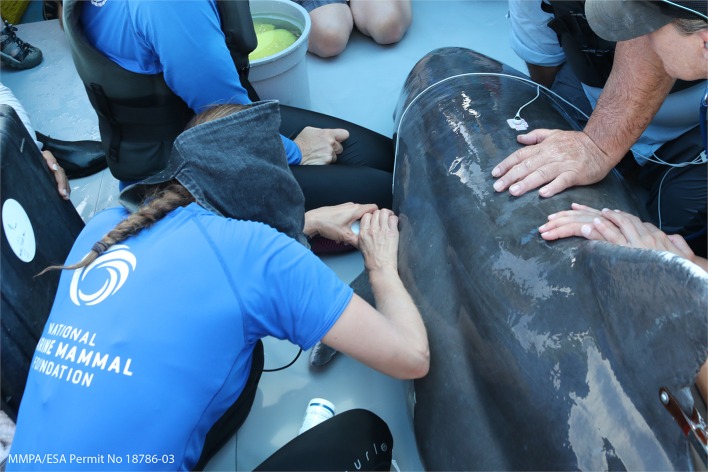
Ultrasound examination of a lymph node on board the veterinary examination and sampling vessel (MMPA ESA Permit No. 18786-03).

Determination of reproductive status (e.g., pregnancy, ovarian activity, or testis size as an indicator of sexual maturity) using diagnostic ultrasound was first applied in Sarasota Bay in 1989 (26, 114). Full-body ultrasound has subsequently been proven to be an invaluable, real-time tool during dolphin health assessments, pioneered by the National Marine Mammal Foundation and the U.S. Navy Marine Mammal Program (Figure 3) (77, 97, 101–103). In addition, pregnancy may also be diagnosed remotely by using blubber biopsy hormone levels (115, 116). In some cases, post-release visual monitoring and photographic-identification (photo-ID) have allowed researchers to track the outcome of the pregnancy (that was determined by ultrasound or remotely) and determine whether a viable calf was produced (82, 116, 117).

**Figure 4:**
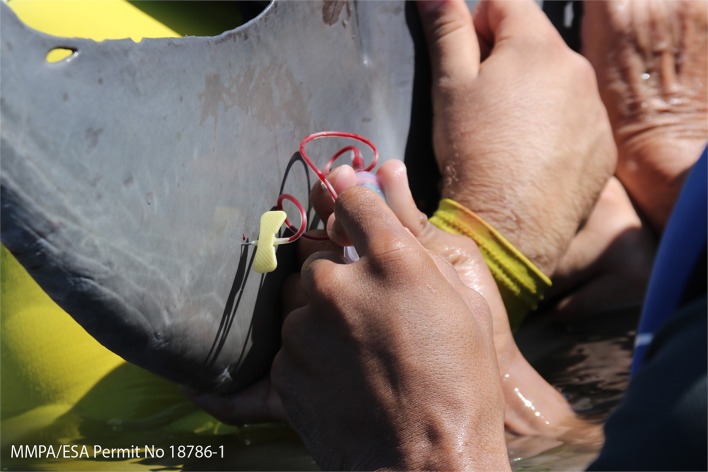
Blood sample collection from the peri-arterial rete on the ventral aspect of the tail fluke (MMPA ESA Permit No. 18786-01).

A holistic approach of including dietary assessment into the health exam along with urinalysis, blood (Figure 4), and blubber sampling can aim to elucidate underlying causes of health abnormalities. In the field, urinary catheterization has enabled comparison with dolphins in managed care and further developed the understanding of the development of renal pathology in dolphins, especially when differences in diets have been considered (91, 97–99). Important ecological perspectives can be obtained from dietary assessments, along with information regarding prey availability and potential shifts in environmental pressure affecting the ecosystem (118–121). Further research is needed in this area to develop a more standardized nutritional status indicator which can integrate multiple measures. This could be used for example to improve understanding of the effects of prey instability or environmental stressors (122).

Electronic tagging technology to assess individual movement or habitat use has rapidly advanced over the past 40 years so that now small satellite-linked tags can be attached via a single-pin to the dorsal fin and have minimal to no long-term effects on the tagged individual (70, 111). These tags provide fine-scale data on individual animal movements for several months, post-release, and can provide additional insight into the cause of health effects identified during the veterinary examination (37, 38, 117). The movement pattern data from these electronic tags can also be used to conduct follow-up monitoring to assess individual animal health, survival, reproductive success, habitat use, and exposure to threats (82).

## Multidisciplinary Approaches for Assessing Population Health

Utilizing a multidisciplinary approach to combine clinical veterinary knowledge with epidemiological analyses and population modeling enables long-term forecasting of population trajectories (36). In UMEs, modeling to estimate mortality based on the number of stranded carcasses can provide insight into the immediate losses to the population (71, 123–125). However, integration of available health information and veterinary interpretation of sublethal, chronic conditions, which are likely to influence long-term survival and reproductive potential, can provide a more accurate interpretation of the likely long-term impacts on the population (36). Aside from impacts from acute events such as oil spills, modeling has been used to simulate the likely population-level consequences from sublethal effects of chronic contaminant exposure (60, 67). Estimation of long-term population effects resulting from mortality or morbidity events is critical to inform restoration or recovery plans, and also to appreciate the magnitude of the impact of UMEs on population numbers or of environmental contaminants on the surrounding ecosystem. Advances in modeling application to marine mammal stock assessments will greatly shape the future of marine mammal conservation.

## Advances in Technology and Considerations for Animal Well-being

Dolphin health assessments provide fine-scale information on individual animals that can be extrapolated to evaluate overall population health. While much can be learned from hands-on health assessments, a major driver of current research is to develop techniques to obtain maximum health assessment information from remote sampling and observations. The methodology for safely handling, sampling, and releasing dolphins is continually evolving to minimize the risk to both the dolphins and researchers, as well as maximize the data collected. However, health assessments are still expensive, logistically challenging, have limited target populations, and there is an inherent risk when handling large animals (126, 127). The development of remote sampling technologies is essential to build upon the data collected during hands-on studies and to expand our ability to efficiently and comprehensively assess the health of dolphin populations beyond nearshore waters, as well as the health of larger, less tractable cetacean species.

**Figure 5:**
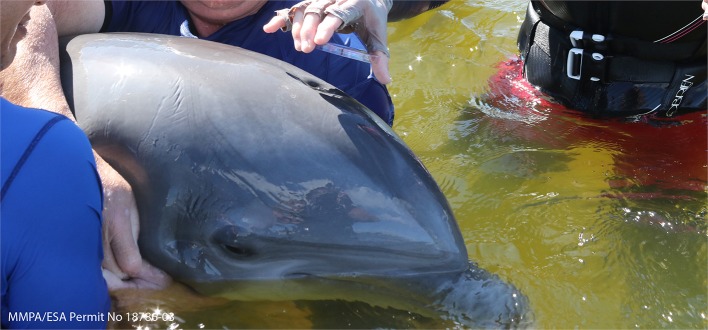
Exhaled breath sample collection for cytology (MMPA ESA Permit No. 18786-03).

Application of new technologies to improve remote sampling opportunities and maximize information obtained from cetacean health assessments is pushing the boundaries of current marine mammal science. Blow samples previously established when in-hand (Figure 5) can now be obtained remotely utilizing UAVs (unmanned aerial vehicles). Drones can be used to obtain aerial images to perform photogrammetry to assess health via body condition in large whales unable to be examined physically (107, 128). Drones can also be used in cetacean disentanglement approaches to provide accurate assessment of exact entanglement points and facilitate more informed decisions on disentanglement methodology. Thermography has been used to assess dolphin dorsal fin temperatures, as a measure of individual health status representing appropriate integumentary thermoregulation (129), and now thermal imaging from drones is being developed to apply to large whale health assessments. Remote temperature assessment will be of increased value in the future when potential climate change impacts could result in cetaceans being exposed to higher or lower environmental temperatures (130–132).

**Figure 6:**
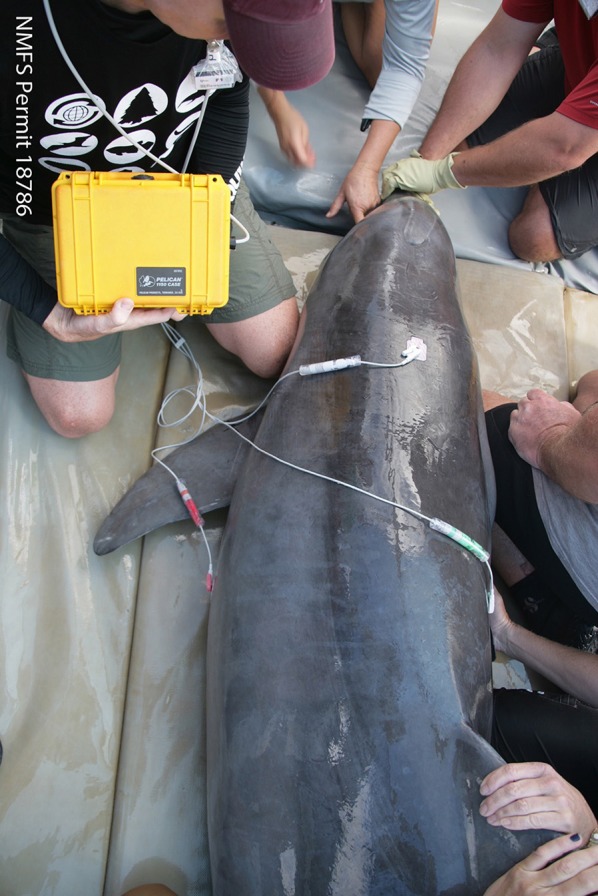
ECG leads attached during sampling and processing to closely monitor the dolphin's heart rate and assess cardiac function (MMPA ESA Permit No. 18786-03).

**Figure 7:**
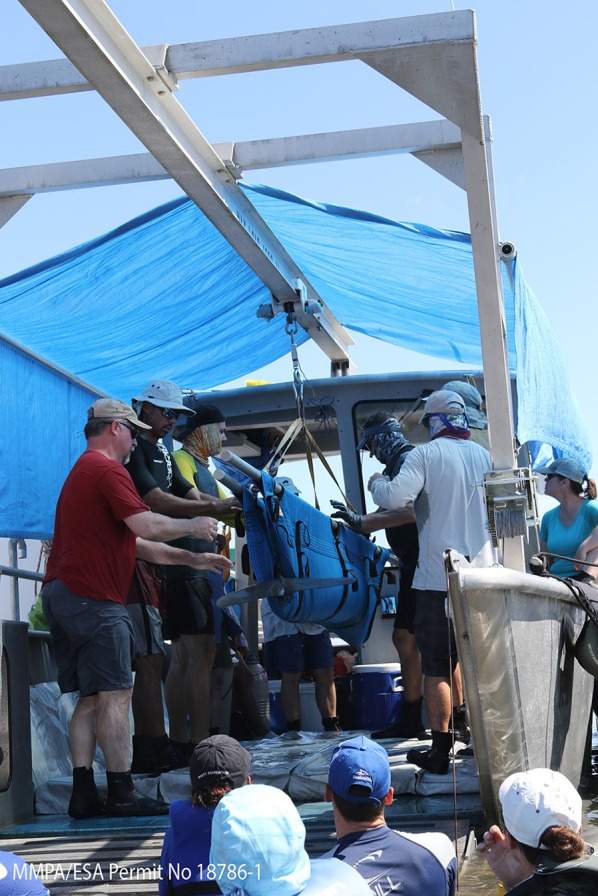
Dolphin suspended in a stretcher for weight measurement via load cell on board the veterinary processing vessel. This image was published with permission of MMPA ESA Permit No. 18786-01 for the identifiable individuals in the image.

Historically, the standard method of estimating the age in dolphin health assessments is via tooth extraction under local anesthesia and counting the growth layer groups present on longitudinal section (17). Dental radiography has been pioneered in an effort to replace the tooth extraction technique, and validation of the technique is ongoing. Bone density assessment has also been explored as a possible aging method, however correlation with age across the entire lifespan was limited (133). A promising new methodology is the use of pectoral flipper radiography to assess bone maturation (134). The dolphin pectoral flipper displays both hyperphalangy and paedomorphosis enabling this method to be applied throughout the entire lifespan due to the predictable chronological osteogenic changes occurring to the metacarpal and phalangeal bones. This non-invasive technology could facilitate age estimation for older animals, replacing tooth extraction.

Additional biological information from remote biopsy dart sampling is expanding on the knowledge gained from each individual sample (135). Currently, sex and population structure of the animal can be determined from genetic analyses of skin (112), and contaminant concentrations and stress and reproductive hormone levels can be measured from blubber biopsy (33, 116, 136). Present efforts are working toward using skin to assess the epigenetics of the individual to give an estimation of age (137). The NMMF are expanding on this even further in line with recent human advancements to provide an indication of biological age (138). This emerging technology could provide a means to assess increased environmental pressure or poor health status (139–141).

Remotely deployed suction cup satellite-linked tags can provide short-term (<24 h) data on bioenergetics, respiratory measures and cardiac data (142, 143). An additional remote tool in development is the use of remotely attached single-pin satellite-linked tags. These techniques will be particularly useful in marine mammals where capture-release is impractical due to size, species intolerance to handling or cost restraints. Future research aims to combine different disciplines to expand scientific knowledge further and inter-species application of new technologies, for example, studying acoustic communication as a proxy to changes in health status (144, 145).

## Discussion

Health assessments with an epidemiological focus can aim to understand the pathophysiology of disease and interpret the demographic, anthropogenic, and ecological pressures contributing to individual disease susceptibility (146). Extrapolating from individual health assessments to accurately understand population health status, requires a strategic epidemiological approach (41, 147, 148). Integration of post-mortem examinations within the health assessment framework can provide additional projections from both diagnostic and scientific perspectives aiming to contribute to identifying the underlying cause of mortality and also predicting the future impacts on the population. Performing pro-active marine mammal health assessment examinations allows an opportunity to examine population health under natural environmental conditions, as opposed to during a mass stranding or UMEs. This baseline knowledge of population health status can aid understanding of post-mortem examinations during UMEs and ultimately aim to drive mitigation strategies for successful conservation and species management. Health assessments are facilitating a pro-active approach to marine mammal conservation in addition to a reactive response to UMEs.

The primary role of wildlife veterinarians is shifting from management of high mortality disease epidemics to preventative management and mitigation of anthropogenic causes of mortality (7). Unlike terrestrial species where mass mortalities garner a lot of public attention, marine species can die in large numbers, and the impact can go unnoticed (149). Sharing information regarding health and threats to local populations can facilitate public interest in coastal and estuarine bottlenose dolphin populations. Increased public awareness and reporting of marine mammals in distress can aid understanding of the current global changes and interactions between humans and wildlife, dictating the efforts required to conserve future marine populations globally.

Continuous monitoring of specific populations over time has the benefit of providing both cross-sectional analyses on an annual basis, as well as longitudinal analysis over several decades. Collecting health data consistently across multiple populations facilitates an understanding of geographic variability, can help to establish reference ranges that are generalizable across populations, and can provide a gradient of stressor exposures for cross-sectional or correlational studies. The combined approaches support a robust framework for epidemiological studies to investigate the causal factors for disease. The collection of decades of archival samples from multiple populations facilitates retrospective studies to discern between sublethal pathogen levels, assess temporal and spatial trends, and elucidate the intricacies of disease susceptibility at a population health level. For example, dolphins in Florida are frequently exposed to various levels of *K. brevis* red tides (150, 151). Examining samples from 1994 to 2003 enabled knowledge of baseline levels of brevetoxin in the dolphin population and demonstrated that dolphin carcasses not associated with large scale mass mortality could also contain comparably high levels of brevetoxins (79). This information is invaluable for future research when the duration and intensity of red tides in Florida appears to be increasing (152, 153).

An additional benefit of long-term studies and sample archives generated by health assessments is the ability to apply new technology and diagnostic tests retrospectively enabling advanced monitoring of health changes over time. Identification of emerging infectious causes of mortality such as cetacean morbillivirus requires continued monitoring of levels of herd immunity over long periods of time (81). Sample archives can be used to establish normal levels and improve understanding of emergence, dynamics, and history of pathogens such as morbillivirus or retrospective analysis of brucella (23, 30, 73). Established baseline data can aid interpretation of normal or increased prevalence of positive antibody titers within the population.

### Stress of Health Assessments (Alternative Perspective)

Prior to considering health assessments for a project, there should be an in-depth discussion of research priorities and if the short-term capturing of individual dolphins is truly the best tool to address the goals of the study. The value of the data obtained from health assessments of free-ranging dolphins needs to be balanced against the potential stress and risk to the individual from the capture, handling, and sampling process (154–157). The stress of capture often influences baseline data such as blood cortisol and aldosterone levels (158). If baseline hormone values are the focus of a study, remote biopsy sampling of blubber can give an accurate indication of baseline stress hormone levels without the elevation caused by the stress of capture (159). However, the adrenal response to a stimulus such as capture may be of interest (35), and capture-release studies enable the evaluation of an individual's hypothalamic pituitary axis and whether or not the animal is capable of mounting an appropriate stress response (75).

In general, the molecular physiological response to the stress of the veterinary examination is well-documented across species; it is transient and the valuable information obtained from the exam typically offsets any acute stress that may be caused. Based on ongoing population monitoring, long-term health consequences of repeated captures have not been found for individuals examined as many as 15 times or more (160). Ensuring capture and restraint are relatively brief and as calm as possible is important, as it has been shown that short holding times do not induce a significant neuroendocrine stress response (154). An experienced team and ongoing training opportunities among organizations, in both managed and free-ranging dolphin populations, promote this high standard of assessment.

Globally, free-ranging dolphins are exposed to a wide range of anthropogenic stressors including environmental contaminants, acoustic shipping disturbances, fisheries interactions, habitat degradation or even loss altogether, exposure to biotoxins, climate disruption, and human interactions (161–168). Variability in the level of anthropogenic stress occurs across geographical locations. Health assessments provide a portal into the individual and population health status to facilitate understanding of ecosystem health, and drive conservation and management decisions. The cost of each individual health assessment is offset by the biological information obtained from each examination. Combining these data with additional stranding information, such as post-mortem examinations and field observations, helps to improve understanding and interpretation of biological health assessment data from a conservation perspective.

### International Perspective

Biologists and veterinarians from around the world have had the opportunity to partake in dolphin health assessments across U.S. waters with the aim of facilitating capacity building for global cetacean conservation (92). Established dolphin health parameter reference ranges enable comparison between international locations in an effort to tease apart the health effects of different stressors. Throughout the majority of the world, health monitoring of dolphins primarily involves post-mortem exams of stranded cetaceans in Europe, physical exams during translocations such as out-of-habitat animals in Asia and South America such as the recent intervention in Bolivia, or during remote biopsy sampling in photo-identification studies in the Mediterranean (169–173).

Increased international collaboration is essential for mitigating conservation crises and aiming to reduce the number of marine mammals becoming extinct such as the recent loss of the Yangtze River dolphin, the baiji (*Lipotes vexillifer*), or the high-risk Mediterranean monk seal (*Monachus monachus*) and vaquita porpoise (*Phocoena sinus*) (4, 174). Knowledge of capture techniques gained from dolphin health assessments in the U.S. has been applied to alternative species conservation approaches, such as with the vaquita, in an effort to temporarily remove animals from a dangerous habitat and relocate them to a protected environment (175). Marine mammal stranding networks exist world-wide with varying capacity dependent on funding, degree of public interest, number of strandings per year, facilities available, and the extent of inter-agency cooperation (176). Sharing knowledge and organizational structure from locations with financial support can aid capacity building in areas where marine mammal stranding networks are currently limited.

From examining dolphin communication to understanding energetics, lung capacity, respiratory metabolomics, and the mechanisms involved in deep diving physiology, health assessments are contributing to advancing scientific knowledge (108, 177–179). Scientists have improved our understanding of dolphin anatomy and physiology by observing natural behavior during health assessments and monitoring activity and behavior post handling. Collaboration among scientists, veterinarians, and biologists at different health assessments enables a synergistic approach to understanding marine mammal health.

Universally, comprehensive cetacean conservation benefits from an integration of all available cetacean assessment techniques; hands on veterinary health assessments, post-mortem examinations, photo-ID surveys, and field observer data. Ideally a collaborative approach among scientists, biologists, fishers, local community members, and government officials would achieve maximum success from a management perspective. Increased discussion will aim to improve future inter-disciplinary approaches and address anthropogenic impacts on marine mammals.

## Conclusion

The advancement of common bottlenose dolphin health assessments, transitioning from initial population assessments to endangered species conservation applications has occurred over several decades, expanding knowledge of marine mammal medicine and science. As veterinary standards for dolphins in human care have evolved, so have the standard protocols for handling and monitoring free-ranging dolphins, and diagnostics such as clinicopathology, ultrasonography, radiography, electrocardiography, respirometry, microbiology, and morphometry (29, 32, 43, 77, 104, 108, 180, 181). Dolphin health assessments are a valuable tool to extrapolate from the individual to understanding both population and ecosystem health. Combining scientific investigation with longitudinal population monitoring over multiple dolphin populations provides information to facilitate informed decisions regarding conservation, regulations, and protection of marine mammals.

Social education regarding the presence, longevity, and residence of bottlenose dolphins also allows the public to engage with the species and appreciate their intrinsic value within the ecosystem; dolphins share the same habitat and are impacted by some of the same stressors as local human communities. Citizen science and public interest peaks during UMEs when multiple carcasses are observed on the beaches in a short time frame. This is an opportunity to engage with people to highlight the environmental pressures faced by these apex predators and explain the anthropogenic impacts on these charismatic species.

An interdisciplinary and interagency approach is needed to fully understand the complexities of the challenges faced by marine mammals. Shifts in the tide of social attitudes, interests, regulations, and funding will have consequences to marine mammal populations both free-ranging as well as managed. Understanding the current global challenges and increasing human and wildlife interactions will dictate the mitigation efforts required to conserve future marine populations. Remaining at the cutting edge of science and advancing the field will aim to facilitate conservation of marine mammal populations for future generations.

## Author Contributions

All authors contributed to the past, present, or future development of dolphin health assessments. AB and RW completed the manuscript writing with significant contributions from LS and CS. All authors provided edits and contributed to the final submitted manuscript.

### Conflict of Interest

JS was employed by the company Dolphin Quest. The remaining authors declare that the research was conducted in the absence of any commercial or financial relationships that could be construed as a potential conflict of interest.

## References

[1] Gulland FMD Hall AJ Is marine mammal health deteriorating? Trends in the global reporting of marine mammal disease. EcoHealth. (2007) 4:135–50. 10.1007/s10393-007-0097-1

[2] Myers N Mittermeier RA Mittermeier CG Da Fonseca GA Kent J. Biodiversity hotspots for conservation priorities. Nature. (2000) 403:853. 10.1038/35002501 10706275

[3] Aguirre AA Ostfeld RS Tabor GM House C Pearl MC Conservation Medicine: Ecological Health in Practice. New York, NY: Oxford University Press (2002).

[4] Reynolds JE Marsh H Ragen TJ Marine mammal conservation. Endang Species Res. (2009) 7:23–8. 10.3354/esr00179

[5] Wikelski M Cooke SJ. Conservation physiology. Trends Ecol Evol. (2006) 21:38–46. 10.1016/j.tree.2005.10.018 16701468

[6] Daszak P Cunningham AA Hyatt AD. Emerging infectious diseases of wildlife–threats to biodiversity and human health. Science. (2000) 287:443–9. 10.1126/science.287.5452.443 10642539

[7] Deem SL Karesh WB Weisman W Putting theory into practice: wildlife health in conservation. Conserv Biol. (2001) 15:1224–33. 10.1046/j.1523-1739.2001.00336.x

[8] Iucn The IUCN Red List of Threatened Species. Version 2019-2. (2019). Available online at: http://www.iucnredlist.org

[9] Taylor BL Martinez M Gerrodette T Barlow J Hrovat YN Lessons from monitoring trends in abundance of marine mammals. Mar Mamm Sci. (2007) 23:157–75. 10.1111/j.1748-7692.2006.00092.x

[10] Davidson AD Boyer AG Kim H Pompa-Mansilla S Hamilton MJ Costa DP. Drivers and hotspots of extinction risk in marine mammals. Proc Natl Acad Sci USA. (2012) 109:3395–400. 10.1073/pnas.1121469109 22308490 PMC3295301

[11] Asper ED Odell DK Bottlenose Dolphin Local Herd Monitoring: Captive Marking, Collection of Biological Data, and Follow-Up Observations of Marked Animals. Final Report to National Marine Fisheries Service, Contract NA79-6A-C-00027. Hubbs/Sea World Research Institute (1980).

[12] Odell DK Asper ED Distribution and movements of freeze-branded bottlenose dolphins in the Indian and Banana Rivers, Florida. In: Leatherwood S Reeves R editors. The Bottlenose Dolphin. Elsevier (1990). p. 515–40.

[13] Solangi MA Dukes GE Atlantic Bottlenose Dolphin, Tursiops Truncatus Herd Studies in the Mississippi Sound, USA: Capture, Freeze Marking and Biological Sampling. Biloxi, MS: Marine Animal Productions (1983).

[14] Irvine B Wells RS Results of attempts to tag atlantic bottlenosed dolphins (*Tursiops truncatus*). Biol Syst. (1972) 13:1–5.

[15] Irvine AB Scott M Wells RS Kaufman JH Movements and activities of the Atlantic bottlenose dolphin, *Tursiops truncatus*, near Sarasota, Florida. Fishery Bull. (1981) 79:135–43.

[16] Wells RS Scott MD Irvine AB The social structure of free-ranging bottlenose dolphins. In: Genoways HH editor. Current Mammalogy. New York, NY: Springer (1987). p. 247–305.

[17] Hohn AA Scott MD Wells RS Sweeney JC Irvine AB Growth layers in teeth from known-age, free-ranging bottlenose dolphins. Mar Mamm Sci. (1989) 5:315–42. 10.1111/j.1748-7692.1989.tb00346.x

[18] Wells RS Scott MD Estimating bottlenose dolphin population parameters from individual identification and capture-release techniques. Rep Int Whal Commission. (1990) 12:407–15.

[19] Duffield DA Wells RS The combined application of chromosome, protein and molecular data for the investigation of social unit structure and dynamics in Tursiops truncatus. In: Hoelzel AR editor. Genetic Ecology of Whales and Dolphins. Cambridge, UK (1991). p. 155–69.

[20] Becker PR Wilkinson DM Lillestolen TI Marine Mammal Health and Stranding Response Program: Program Development Plan. NOAA Technical Memorandum NMFS-OPR-94-2, National Oceanic and Atmospheric Administration, Silver Spring, MD (1994).

[21] Geraci JR Clinical Investigation of the 1987-88 Mass Mortality of Bottlenose Dolphins Along the US Central and South Atlantic Coast. Final report to National Marine Fisheries Service and US Navy Office of Naval Research and Marine Mammal Commission (1989).

[22] Lipscomb TP Schulman FY Moffett D Kennedy S. Morbilliviral disease in Atlantic bottlenose dolphins (*Tursiops truncatus*) from the 1987-1988 epizootic. J Wildl Dis. (1994) 30:567–71. 10.7589/0090-3558-30.4.567 7760492

[23] Duignan PJ House C Odell DK Wells RS Hansen LJ Walsh MT Morbillivirus infection in bottlenose dolphins: evidence for recurrent epizootics in the western Atlantic and Gulf of Mexico. Mar Mamm Sci. (1996) 12:499–515. 10.1111/j.1748-7692.1996.tb00063.x

[24] Hansen LJ Wells RS Bottlenose Dolphin Health Assessment: Field Report on Sampling Near Beaufort, North Carolina, During July, 1995. NOAA Technical Memorandum NMFS-SEFSC-382 (1996).

[25] Mclellan WA Friedlaender AS Mead JG Potter CW Pabst DA Analysing 25 years of bottlenose dolphin (*Tursiops truncatus*) strandings along the Atlantic coast of the USA: do historic records support the coastal migratory stock hypothesis? J Cetacean Res Manag. (2002) 4:297–304. Available online at: https://pdfs.semanticscholar.org/1ec1/4350253d6e3276a4d1d0021959b20fc59706.pdf

[26] Sweeney JC Stone LR Townsend FI Casper D Hansen L Population Health Assessment on Tursiops truncatus From Matagorda Bay, Texas, Following a Mortality Event, 1992. NOAA-NMFS, SEFSC Contribution MIA-92/93-41, 10 pp + appendices (1992).

[27] Schwacke LH Twiner MJ De Guise S Balmer BC Wells RS Townsend FI. Eosinophilia and biotoxin exposure in bottlenose dolphins (*Tursiops truncatus*) from a coastal area impacted by repeated mortality events. Environ Res. (2010) 110:548–55. 10.1016/j.envres.2010.05.003 20537621

[28] Lohoefener R Hoggard W Mullin K Ford R Benigno J Studies of Mississippi Sound Bottlenose Dolphins: Estimates of Bottlenose Dolphin density in Mississippi Sound From Small Boat Surveys. Pascagoula, MS: National Marine Fisheries Service (1990).

[29] Wells RS Rhinehart HL Hansen LJ Sweeney JC Townsend FI Stone R Bottlenose dolphins as marine ecosystem sentinels: developing a health monitoring system. EcoHealth. (2004) 1:246–54. 10.1007/s10393-004-0094-6

[30] Rowles T Schwacke L Wells R Saliki J Hansen L Hohn A Evidence of susceptibility to morbillivirus infection in cetaceans from the United States. Mar Mamm Sci. (2011) 27:1–19. 10.1111/j.1748-7692.2010.00393.x

[31] Hansen LJ Schwacke LH Mitchum GB Hohn AA Wells RS Zolman ES. Geographic variation in polychorinated biphenyl and organochlorine pesticide concentrations in the blubber of bottlenose dolphins from the US Atlantic coast. Sci Total Environ. (2004) 319:147–72. 10.1016/S0048-9697(03)00371-1 14967508

[32] Schwacke LH Hall AJ Townsend FI Wells RS Hansen LJ Hohn AA. Hematologic and serum biochemical reference intervals for free-ranging common bottlenose dolphins (*Tursiops truncatus*) and variation in the distributions of clinicopathologic values related to geographic sampling site. Am J Vet Res. (2009) 70:973–85. 10.2460/ajvr.70.8.973 19645578

[33] Kucklick J Schwacke L Wells R Hohn A Guichard A Yordy J. Bottlenose dolphins as indicators of persistent organic pollutants in the western North Atlantic Ocean and northern Gulf of Mexico. Environ Sci Cons. (2011) 45:4270–7. 10.1021/es1042244 21526819

[34] Bossart GD Fair P Schaefer AM Reif JS. Health and Environmental Risk Assessment Project for bottlenose dolphins *Tursiops truncatus* from the southeastern USA. I Infectious diseases. Dis Aquat Org. (2017) 125:141–53. 10.3354/dao03142 28737159

[35] Schwacke LH Smith CR Townsend FI Wells RS Hart LB Balmer BC Health of common bottlenose dolphins (*Tursiops truncatus*) in Barataria Bay, Louisiana, following the deepwater horizon oil spill. Environ Sci Technol. (2014) 48:93–103. 10.1021/es403610f 24350796

[36] Schwacke LH Thomas L Wells RS Mcfee WE Hohn AA Mullin KD Quantifying injury to common bottlenose dolphins from the Deepwater Horizon oil spill using an age-, sex-and class-structured population model. Endang Species Res. (2017) 33:265–79. 10.3354/esr00777

[37] Mullin KD Mcdonald T Wells RS Balmer BC Speakman T Sinclair C. Density, abundance, survival, and ranging patterns of common bottlenose dolphins (*Tursiops truncatus*) in Mississippi Sound following the Deepwater Horizon oil spill. PLoS ONE. (2017) 12:e0186265. 10.1371/journal.pone.0186265 29053728 PMC5650146

[38] Balmer B Zolman E Rowles T Smith C Townsend F Fauquier D. Ranging patterns, spatial overlap, and association with dolphin morbillivirus exposure in common bottlenose dolphins (*Tursiops truncatus*) along the Georgia, USA coast. Ecol Evol. (2018) 8:12890–4. 10.1002/ece3.4727 30619591 PMC6308875

[39] Garrison LP Hohn AA Hansen LJ Seasonal Movements of Atlantic Common Bottlenose Dolphin Stocks Based on Tag Telemetry Data. Southeast Fisheries Science Center, Protected Resources and Biodiversity Division (2017). p. 75.

[40] Scott GP Burn DM Hansen LJ The Dolphin Dieoff: Long-Term Effects and Recovery of the Population. IEEE (1998). p. 819–23.

[41] Rowles TK Schwacke LH Hall AJ Barbieri M Population health assessment study design. In: Gulland FMD Whitman KL Dierauf LA editors. CRC Handbook of Marine Mammal Medicine. CRC Press (2018). p. 813–22.

[42] Goldstein JD Reese E Reif JS Varela RA Mcculloch SD Defran RH. Hematologic, biochemical, and cytologic findings from apparently healthy atlantic bottlenose dolphins (*Tursiops truncatus*) Inhabiting the Indian River Lagoon, Florida, USA. J Wildl Dis. (2006) 42:447–54. 10.7589/0090-3558-42.2.447 16870874

[43] Reif JS Fair PA Adams J Joseph B Kilpatrick DS Sanchez R. Evaluation and comparison of the health status of Atlantic bottlenose dolphins from the Indian River Lagoon, Florida, and Charleston, South Carolina. J Am Vet Med Ass. (2008) 233:299–307. 10.2460/javma.233.2.299 18627240

[44] Reif JS Schaefer AM Bossart GD Fair PA. Health and environmental risk assessment project for bottlenose dolphins *Tursiops truncatus* from the southeastern USA. II Environmental aspects. Dis Aquat Org. (2017) 125:155–66. 10.3354/dao03143 28737160

[45] Litz JA Baran MA Bowen-Stevens SR Carmichael RH Colegrove KM Garrison LP. Review of historical unusual mortality events (UMEs) in the Gulf of Mexico (1990-2009): providing context for the multi-year northern Gulf of Mexico cetacean UME declared in 2010. Dis Aquat Org. (2014) 112:161–75. 10.3354/dao02807 25449327

[46] Twiner MJ Flewelling LJ Fire SE Bowen-Stevens SR Gaydos JK Johnson CK. Comparative analysis of three brevetoxin-associated bottlenose dolphin (*Tursiops truncatus*) mortality events in the Florida panhandle region (USA). PLoS ONE. (2012) 7:e42974. 10.1371/journal.pone.0042974 22916189 PMC3419745

[47] Gulland FMD Haulena M Fauquier D Langlois G Lander ME Zabka T. Domoic acid toxicity in Californian sea lions (*Zalophus californianus*): clinical signs, treatment and survival. Vet Rec. (2002) 150:475–80. 10.1136/vr.150.15.475 11995679

[48] Balmer BC Wells RS Nowacek SM Nowacek DP Schwacke LH Mclellan WA 157 Seasonal abundance and distribution patterns of common bottlenose dolphins (*Tursiops truncatus*) near St. Joseph Bay, Florida, USA. J Cetacean Res Manage. (2008) 10:157–67. Available online at: https://uncw.edu/mmsp/documents/balmer_et_al_2008.pdf

[49] Vollmer NL Rosel PE A review of common bottlenose dolphins (*Tursiops truncatus truncatus*) in the northern Gulf of Mexico: population biology, potential threats, and management. Southeastern Natural. (2013) 12:1–44. 10.1656/058.012.m601

[50] Balmer B Mcdonald T Hornsby F Adams J Allen J Barleycorn A Long-term trends in a northern Gulf of Mexico common bottlenose dolphin (*Tursiops truncatus*) population in the wake of the Deepwater Horizon oil spill. J Cetacean Res Manage. (2018) 18:1–9. Available online at: https://www.researchgate.net/profile/Brian_Balmer/publication/328076117_Long-term_trends_in_a_northern_Gulf_of_Mexico_common_bottlenose_dolphin_Tursiops_truncatus_population_in_the_wake_of_the_Deepwater_Horizon_oil_spill/links/5bb6324e4585159e8d86662f/Long-term-trends-in-a-northern-Gulf-of-Mexico-common-bottlenose-dolphin-Tursiops-truncatus-population-in-the-wake-of-the-Deepwater-Horizon-oil-spill.pdf

[51] Fauquier D Kinsel M Dailey M Sutton G Stolen M Wells R. Prevalence and pathology of lungworm infection in bottlenose dolphins *Tursiops truncatus* from southwest Florida. Dis Aquat Org. (2009) 88:85–90. 10.3354/dao02095 20183968

[52] Bogomolni AL Pugliares KR Sharp SM Patchett K Harry CT Larocque JM. Mortality trends of stranded marine mammals on Cape Cod and southeastern Massachusetts, USA, 2000 to 2006. Dis Aquat Org. (2010) 88:143–55. 10.3354/dao02146 20225675

[53] Wells RS Allen JB Lovewell G Gorzelany J Delynn RE Fauquier DA Carcass-recovery rates for resident bottlenose dolphins in Sarasota Bay, Florida. Mar Mamm Sci. (2015) 31:355–68. 10.1111/mms.12142

[54] Moore MJ Mclellan WA Daoust PY Bonde RK Knowlton AR Right whale mortality: a message from the dead to the living. In: Kraus SD Rolland R editors. The Urban Whale: North Atlantic Right Whales at the Crossroads. Cambridge, MA: Harvard University Press (2007).

[55] Martineau D Béland P Desjardins C Lagacé A Levels of organochlorine chemicals in tissues of beluga whales (*Delphinapterus leucas*) from the St. Lawrence Estuary, Quebec, Canada. Environ Contamin Toxic. (1987) 16:137–47. 10.1007/BF01055795

[56] Kuehl DW Haebler R. Organochlorine, organobromine, metal, and selenium residues in bottlenose dolphins (*Tursiops truncatus*) collected during an unusual mortality event in the Gulf of Mexico, 1990. Arch Environ Contam Toxicol. (1995) 28:494–9. 10.1007/BF00211632 7755403

[57] Wells RS Tornero V Borrell A Aguilar A Rowles TK Rhinehart HL. Integrating life-history and reproductive success data to examine potential relationships with organochlorine compounds for bottlenose dolphins (*Tursiops truncatus*) in Sarasota Bay, Florida. Sci Total Environ. (2005) 349:106–19. 10.1016/j.scitotenv.2005.01.010 16198673

[58] Yordy JE Pabst DA Mclellan WA Wells RS Rowles TK Kucklick JR. Tissue-specific distribution and whole-body burden estimates of persistent organic pollutants in the bottlenose dolphin (*Tursiops truncatus*). Environ Toxicol Chem. (2010) 29:1263–73. 10.1002/etc.152 20821568

[59] Jepson PD Law RJ. Persistent pollutants, persistent threats. Science. (2016) 352:1388–9. 10.1126/science.aaf9075 27313021

[60] Schwacke LH Voit EO Hansen LJ Wells RS Mitchum GB Hohn AA. Probabilistic risk assessment of reproductive effects of polychlorinated biphenyls on bottlenose dolphins (*Tursiops truncatus*) from the southeast United States coast. Environ Toxicol Chem. (2002) 21:2752–64. 10.1002/etc.5620211232 12463575

[61] Reijnders PJ. Reproductive failure in common seals feeding on fish from polluted coastal waters. Nature. (1986) 324:456–7. 10.1038/324456a0 3785423

[62] Ross PS Ellis G Ikonomou M Barrett-Lennard L Addison R High PCB concentrations in free-ranging Pacific killer whales, *Orcinus orca*: effects of age, sex and dietary preference. Mar Poll Bull. (2000) 40:504–15. 10.1016/S0025-326X(99)00233-7

[63] Reddy ML Reif J Bachand A Ridgway S. Opportunities for using Navy marine mammals to explore associations between organochlorine contaminants and unfavorable effects on reproduction. Sci Tot Environ. (2001) 274:171–82. 10.1016/S0048-9697(01)00741-0 11453294

[64] Hall AJ Hugunin K Deaville R Law RJ Allchin CR Jepson PD. The risk of infection from polychlorinated biphenyl exposure in the harbor porpoise (*Phocoena phocoena*): a case–control approach. Environ Health Persp. (2006) 114:704–11. 10.1289/ehp.8222 16675424 PMC1459923

[65] Mori C Morsey B Levin M Nambiar PR De Guise S. Immunomodulatory effects of *in vitro* exposure to organochlorines on T-cell proliferation in marine mammals and mice. J Tox Environ Health A. (2006) 69:283–302. 10.1080/15287390500227472 16407088

[66] Schwacke LH Zolman ES Balmer BC De Guise S George RC Hoguet J. Anaemia, hypothyroidism and immune suppression associated with polychlorinated biphenyl exposure in bottlenose dolphins (*Tursiops truncatus*). Proc Biol Sci. (2012) 279:48–57. 10.1098/rspb.2011.0665 21613298 PMC3223648

[67] Hall AJ Mcconnell BJ Rowles TK Aguilar A Borrell A Schwacke L. Individual-based model framework to assess population consequences of polychlorinated biphenyl exposure in bottlenose dolphins. Environ Health Persp. (2005) 114:60–4. 10.1289/ehp.8053 16818247 PMC1874180

[68] Balmer BC Ylitalo GM Mcgeorge LE Baugh KA Boyd D Mullin KD. Persistent organic pollutants (POPs) in blubber of common bottlenose dolphins (*Tursiops truncatus*) along the northern Gulf of Mexico coast, USA. Sci Total Environ. (2015) 527:306–12. 10.1016/j.scitotenv.2015.05.016 25965044

[69] Balmer JE Ylitalo GM Rowles TK Mullin KD Wells RS Townsend FI. Persistent organic pollutants (POPs) in blood and blubber of common bottlenose dolphins (*Tursiops truncatus*) at three northern Gulf of Mexico sites following the Deepwater Horizon oil spill. Sci Total Environ. (2018) 621:130–7. 10.1016/j.scitotenv.2017.11.209 29179068

[70] Balmer BC Wells RS Howle LE Barleycorn AA Mclellan WA Ann Pabst D Advances in cetacean telemetry: a review of single-pin transmitter attachment techniques on small cetaceans and development of a new satellite-linked transmitter design. Mar Mamm Sci. (2014) 30:656–73. 10.1111/mms.12072

[71] Takeshita R Sullivan L Smith C Collier T Hall A Brosnan T The Deepwater Horizon oil spill marine mammal injury assessment. Endanger Species Res. (2017) 33:95–106. 10.3354/esr00808

[72] Wallace BP Brosnan T Mclamb D Rowles T Ruder E Schroeder B Effects of the Deepwater Horizon oil spill on protected marine species. Endang Species Res. (2017) 33:1–7. 10.3354/esr00789

[73] Venn-Watson S Colegrove KM Litz J Kinsel M Terio K Saliki J. Adrenal gland and lung lesions in Gulf of Mexico common bottlenose dolphins (*Tursiops truncatus*) found dead following the Deepwater Horizon oil spill. PLoS ONE. (2015) 10:e0126538. 10.1371/journal.pone.0126538 25992681 PMC4439104

[74] De Guise S Levin M Gebhard E Jasperse L Hart LB Smith CR Changes in immune functions in bottlenose dolphins in the northern Gulf of Mexico associated with the Deepwater Horizon oil spill. Endang Species Res. (2017) 33:291–303. 10.3354/esr00814

[75] Smith CR Rowles TK Hart LB Townsend FI Wells RS Zolman ES Slow recovery of Barataria Bay dolphin health following the Deepwater Horizon oil spill (2013-2014), with evidence of persistent lung disease and impaired stress response. Endang Species Res. (2017) 33:127–42. 10.3354/esr00778

[76] Venn-Watson S Garrison L Litz J Fougeres E Mase B Rappucci G. Demographic clusters identified within the northern Gulf of Mexico common bottlenose dolphin (*Tursiops truncates*) unusual mortality event: January 2010-June 2013. PLoS ONE. (2015) 10:e0117248. 10.1371/journal.pone.0117248 25671657 PMC4324990

[77] Smith CR Solano M Lutmerding BA Johnson SP Meegan JM Le-Bert CR. Pulmonary ultrasound findings in a bottlenose dolphin *Tursiops truncatus* population. Dis Aquat Organ. (2012) 101:243–55. 10.3354/dao02537 23324421

[78] Venn-Watson S Daniels R Smith C. Thirty year retrospective evaluation of pneumonia in a bottlenose dolphin *Tursiops truncatus* population. Dis Aquat Org. (2012) 99:237–42. 10.3354/dao02471 22832722

[79] Fire SE Fauquier D Flewelling LJ Henry M Naar J Pierce R Brevetoxin exposure in bottlenose dolphins (*Tursiops truncatus*) associated with *Karenia brevis* blooms in Sarasota Bay, Florida. Mar Biol. (2007) 152:827–34. 10.1007/s00227-007-0733-x

[80] Colegrove KM Venn-Watson S Litz J Kinsel MJ Terio KA Fougeres E. Fetal distress and *in utero* pneumonia in perinatal dolphins during the Northern Gulf of Mexico unusual mortality event. Dis Aquat Org. (2016) 119:1–16. 10.3354/dao02969 27068499

[81] Fauquier DA Litz J Sanchez S Colegrove K Schwacke LH Hart L Evaluation of morbillivirus exposure in cetaceans from the northern Gulf of Mexico 2010-2014. Endang Species Res. (2017) 33:211–20. 10.3354/esr00772

[82] Lane SM Smith CR Mitchell J Balmer BC Barry KP Mcdonald T. Reproductive outcome and survival of common bottlenose dolphins sampled in Barataria Bay, Louisiana, USA, following the Deepwater Horizon oil spill. Proc Biol Sci. (2015) 282:20151944. 10.1098/rspb.2015.1944 26538595 PMC4650159

[83] Mcdonald TL Hornsby FE Speakman TR Zolman ES Mullin KD Sinclair C Survival, density, and abundance of common bottlenose dolphins in Barataria Bay (USA) following the Deepwater Horizon oil spill. Endang Species Res. (2017) 33:193–209. 10.3354/esr00806

[84] Monson DH Doak DF Ballachey BE Johnson A Bodkin JL. Long-term impacts of the Exxon Valdez oil spill on sea otters, assessed through age-dependent mortality patterns. Proc Nat Acad Sci USA. (2000) 97:6562–7. 10.1073/pnas.120163397 10823920 PMC18659

[85] Bodkin JL Ballachey BE Dean T Fukuyama AK Jewett S Mcdonald L Sea otter population status and the process of recovery from the 1989 ‘Exxon Valdez'oil spill. Mar Ecol Prog Ser. (2002) 241:237–54. 10.3354/meps241237

[86] Matkin C Saulitis E Ellis G Olesiuk P Rice S Ongoing population-level impacts on killer whales *Orcinus orca* following the'Exxon Valdez'oil spill in Prince William Sound, Alaska. Mar Ecol Prog Ser. (2008) 356:269–81. 10.3354/meps07273

[87] Helm RC Costa DP Debruyn TD O'shea TJ Wells RS Williams TM Overview of effects of oil spills on marine mammals. In: Fingus MF editor. Handbook of Oil Spill Science and Technology. John Wiley & Sons, Inc (2015). p. 455–75.

[88] Asper ED Techniques of live capture of smaller Cetacea. J Fish Res Board Can. (1975) 32:1191–6. 10.1139/f75-138

[89] Loughlin T Cunningham L Gales N Wells RS Boyd I Marking and capturing. In: Boyd IL Don Bowen W Iverson SJ editors. Marine Mammal Ecology and Conservation: A Handbook of Techniques. Oxford: Oxford University Press (2010). p. 16–41.

[90] Klatsky LJ Wells RS Sweeney JC Offshore bottlenose dolphins (*Tursiops truncatus*): movement and dive behavior near the Bermuda Pedestal. J Mammal. (2007) 88:59–66. 10.1644/05-MAMM-A-365R1.1

[91] Townsend FI Smith CR Rowles T Health assessment of bottlenose dolphins in capture-release studies. In: Gulland FMD Whitman KL Dierauf LA editors. CRC Handbook of Marine Mammal Medicine. CRC Press (2018). p. 823–33.

[92] Wells RS. Learning from nature: bottlenose dolphin care and husbandry. Zoo Biol. (2009) 28:635–51. 10.1002/zoo.20252 19434729

[93] Ridgway SH. Medical care of marine mammals. J Am Vet Med Ass. (1965) 147:1077–85. 5879912

[94] Kellar NM Trego ML Chivers SJ Archer FI Pregnancy patterns of pantropical spotted dolphins (*Stenella attenuata*) in the eastern tropical Pacific determined from hormonal analysis of blubber biopsies and correlations with the purse-seine tuna fishery. Mar Biol. (2013) 160:3113–24. 10.1007/s00227-013-2299-0

[95] Rossman S Barros NB Ostrom PH Stricker CA Hohn AA Gandhi H Retrospective analysis of bottlenose dolphin foraging: a legacy of anthropogenic ecosystem disturbance. Mar Mamm Sci. (2013) 29:705–18. 10.1111/j.1748-7692.2012.00618.x

[96] Boggs ASP Schock TB Schwacke LH Galligan TM Morey JS Mcfee WE. Rapid and reliable steroid hormone profiling in *Tursiops truncatus* blubber using liquid chromatography tandem mass spectrometry (LC-MS/MS). Analyt Bioanalyt Chem. (2017) 409:5019–29. 10.1007/s00216-017-0446-z 28631158 PMC5629977

[97] Smith CR Venn-Watson S Wells RS Johnson SP Maffeo N Balmer BC. Comparison of nephrolithiasis prevalence in two bottlenose dolphin (*Tursiops truncatus*) populations. Front Endocrinol. (2013) 4:145. 10.3389/fendo.2013.00145 24137158 PMC3797464

[98] Smith CR Poindexter JR Meegan JM Bobulescu IA Jensen ED Venn-Watson S. Pathophysiological and physicochemical basis of ammonium urate stone formation in dolphins. J Urol. (2014) 192:260–6. 10.1016/j.juro.2014.01.008 24518786 PMC4330087

[99] Ardente A Wells R Smith C Walsh M Jensen E Schmitt T Dietary cation–anion difference may explain why ammonium urate nephrolithiasis occurs more frequently in common bottlenose dolphins (*Tursiops truncatus*) under human care than in free-ranging common bottlenose dolphins. J Anim Sci. (2017) 95:1396–406. 10.2527/jas.2016.1113 28380506

[100] Sweeney JC Ridgway SH. Procedures for the clinical management of small cetaceans. J Am Vet Med Assoc. (1975) 167:540–5. 1176341

[101] Smith CR Jensen ED Blankenship BA Greenberg M D'agostini DA Pretorius DH. Fetal omphalocele in a common bottlenose dolphin (*Tursiops truncatus*). J Zoo Wildlife Med. (2013) 44:87–92. 10.1638/1042-7260-44.1.87 23505707

[102] Seitz KE Smith CR Marks SL Venn-Watson SK Ivancic M. Liver ultrasonography in dolphins: use of ultrasonography to establish a technique for hepatobiliary imaging and to evaluate metabolic disease-associated liver changes in bottlenose dolphins (*Tursiops truncatus*). J Zoo Wildl Med. (2016) 47:1034–43. 10.1638/2015-0173.1 28080913

[103] Martony ME Ivancic M Gomez FM Meegan JM Nollens HH Schmitt TL. Establishing marginal lymph node ultrasonographic characteristics in healthy bottlenose dolphins (*Tursiops truncatus*). J Zoo Wildl Med. (2017) 48:961–71. 10.1638/2016-0251.1 29297828

[104] Harms CA Jensen ED Townsend FI Hansen LJ Schwacke LH Rowles TK. Electrocardiograms of bottlenose dolphins (*Tursiops truncatus*) out of water: habituated collection versus wild postcapture animals. J Zoo Wildl Med. (2013) 44:972–81. 10.1638/2013-0093.1 24450057

[105] Read A Wells R Hohn A Scott M Patterns of growth in wild bottlenose dolphins, *Tursiops truncatus*. J Zool. (1993) 231:107–23. 10.1111/j.1469-7998.1993.tb05356.x

[106] Houser DS Finneran JJ. Variation in the hearing sensitivity of a dolphin population determined through the use of evoked potential audiometry. J Acoutic Soc Am. (2006) 120:4090–9. 10.1121/1.2357993 17225435

[107] Apprill A Miller CA Moore MJ Durban JW Fearnbach H Barrett-Lennard LG. Extensive core microbiome in drone-captured whale blow supports a framework for health monitoring. mSystems. (2017) 2:e00119–17. 10.1128/mSystems.00119-17 29034331 PMC5634792

[108] Fahlman A Brodsky M Wells R Mchugh K Allen J Barleycorn A. Field energetics and lung function in wild bottlenose dolphins, *Tursiops truncatus*, in Sarasota Bay Florida. R Soc Open Sci. (2018) 5:171280. 10.1098/rsos.171280 29410836 PMC5792913

[109] Buck JD Wells RS Rhinehart HL Hansen LJ. Aerobic microorganisms associated with free-ranging bottlenose dolphins in coastal Gulf of Mexico and Atlantic Ocean waters. J Wildl Dis. (2006) 42:536–44. 10.7589/0090-3558-42.3.536 17092884

[110] Wells RS. Identification methods. In: Würsig B, Thewissen JGM, Kovacs K, eitors. Encyclopedia of Marine Mammals. 3rd ed. San Diego, CA: Academic Press (2018). p. 503–9.

[111] Scott MD Wells RS Irvine AB Mate BR Tagging and marking studies on small cetaceans. In: Appel MJ editor. The Bottlenose Dolphin. New York, NY: Elsevier (1990). p. 489–514.

[112] Rosel PE PCR-based sex determination in Odontocete cetaceans. Conserv Genet. (2003) 4:647–9. 10.1023/A:1025666212967

[113] Schwacke L Hall AJ Wells RS Bossart GD Fair P Hohn AA Health and risk assessment for bottlenose dolphin (*Tursiops truncatus*) populations along the southeast United States coast: current status and future plans. Paper SC/56/E20 Presented to the IWC Scientific Committee. Sorrento (2004).

[114] Wells RS Smith CR Sweeney JC Townsend FI Fauquier DA Stone R Fetal survival of common bottlenose dolphins (*Tursiops truncatus*) in Sarasota Bay, Florida. Aquat Mamm. (2014) 40:252 10.1578/AM.40.3.2014.252

[115] Kellar NM Trego ML Marks CI Dizon AE Determining pregnancy from blubber in three species of delphinids. Mar Mamm Sci. (2006) 22:1–16. 10.1111/j.1748-7692.2006.00001.x

[116] Kellar NM Speakman TR Smith CR Lane SM Balmer BC Trego ML Low reproductive success rates of common bottlenose dolphins *Tursiops truncatus* in the northern Gulf of Mexico following the Deepwater Horizon disaster (2010-2015). Endang Species Res. (2017) 33:143–58. 10.3354/esr00775

[117] Wells RS Schwacke LH Rowles TK Balmer BC Zolman E Speakman T Ranging patterns of common bottlenose dolphins *Tursiops truncatus* in Barataria Bay, Louisiana, following the Deepwater Horizon oil spill. Endang Species Res. (2017) 33:159–80. 10.3354/esr00732

[118] Heithaus MR Dill LM Food availability and tiger shark predation risk influence bottlenose dolphin habitat use. Ecology. (2002) 83:480–91. 10.1890/0012-9658(2002)083[0480:FAATSP]2.0.CO;2

[119] Mccabe EJB Gannon DP Barros NB Wells RS Prey selection by resident common bottlenose dolphins (*Tursiops truncatus*) in Sarasota Bay, Florida. Mar Biol. (2010) 157:931–42. 10.1007/s00227-009-1371-2

[120] Santos MB German I Correia D Read FL Cedeira JM Caldas M Long-term variation in common dolphin diet in relation to prey abundance. Mar Ecol Prog Ser. (2013) 481:249–68. 10.3354/meps10233

[121] Wells R Mchugh KA Douglas DC Shippee S Berens Mccabe EJ Barros NB. Evaluation of potential protective factors against metabolic syndrome in bottlenose dolphins: feeding and activity patterns of dolphins in Sarasota Bay, Florida. Front Endocrinol. (2013) 4:139. 10.3389/fendo.2013.00139 24133483 PMC3794307

[122] Jodice PGR Roby DD Turco KR Suryan RM Irons DB Piatt JF Assessing the nutritional stress hypothesis: relative influence of diet quantity and quality on seabird productivity. Mar Ecol Prog Ser. (2006) 325:267–79. 10.3354/meps325267

[123] Hohn AA Thomas L Carmichael RH Litz J Clemons-Chevis C Shippee SF Assigning stranded bottlenose dolphins to source stocks using stable isotope ratios following the Deepwater Horizon oil spill. Endang Species Res. (2017) 33:235–52. 10.3354/esr00783

[124] Rosel PE Wilcox LA Sinclair C Speakman TR Tumlin MC Litz JA Genetic assignment to stock of stranded common bottlenose dolphins in southeastern Louisiana after the Deepwater Horizon oil spill. Endang Species Res. (2017) 33:221–34. 10.3354/esr00780

[125] Thomas L Booth CG Rosel PE Hohn A Litz J Schwacke LH Where were they from? Modelling the source stock of dolphins stranded after the Deepwater Horizon oil spill using genetic and stable isotope data. Endang Species Res. (2017) 33:253–64. 10.3354/esr00754

[126] Wilson RP Mcmahon CR Measuring devices on wild animals: what constitutes acceptable practice? Frontiers Ecol Environ. (2006) 4:147–54. 10.1890/1540-9295(2006)0040147:MDOWAW2.0.CO;2

[127] Balmer BC Wells RS Schwacke LH Schwacke JH Danielson B George RC Integrating multiple techniques to identify stock boundaries of common bottlenose dolphins (*Tursiops truncatus*). Aquat Conserv Mar Freshw Ecosyst. (2014) 24:511–21. 10.1002/aqc.2357

[128] Pirotta V Smith A Ostrowski M Russell D Jonsen ID Grech A An economical custom-built drone for assessing whale health. Front Mar Sci. (2017) 4:425 10.3389/fmars.2017.00425

[129] Barbieri MM Mclellan WA Wells RS Blum JE Hofmann S Gannon J Using infrared thermography to assess seasonal trends in dorsal fin surface temperatures of free-swimming bottlenose dolphins (*Tursiops truncatus*) in Sarasota Bay, Florida. Mar Mamm Sci. (2010) 26:53–66. 10.1111/j.1748-7692.2009.00319.x

[130] Learmonth JA Macleod CD Santos MB Pierce GJ Crick H Robinson R Potential effects of climate change on marine mammals. Oceanogr Mar Biol. (2006) 44:431 10.1201/9781420006391.ch8

[131] Burek KA Gulland FMD O'hara TM. Effects of climate change on Arctic marine mammal health. Ecol Appl. (2008) 18:126–34. 10.1890/06-0553.1 18494366

[132] Edwards HH Potential impacts of climate change on warmwater megafauna: the Florida manatee example (*Trichechus manatus latirostris*). Clim Change. (2013) 121:727–38. 10.1007/s10584-013-0921-2

[133] Powell JWB Duffield DA Kaufman JJ Mcfee W Bone density cannot accurately predict age in the common bottlenose dolphin, *Tursiops truncatus*. Mar Mam Sci. (2019) 35:1597–602. 10.1111/mms.12591

[134] García-Párraga D Schmitt TL Jensen E Use of radiographic parameters for age estimation in bottlenose dolphins (*Tursiops truncatus*). In: International Association of Aquatic Animal Health Conference Proceedings. (2011).

[135] Weller DW Cockcroft VG Würsig B Lynn SK Fertl D Behavioral responses of bottlenose dolphins to remote biopsy sampling and observations of surgical biopsy wound healing. Aquat Mamm. (1997) 23:49–58.

[136] Boggs ASP Ragland JM Zolman ES Schock TB Morey JS Galligan TM. Remote blubber sampling paired with liquid chromatography tandem mass spectrometry for steroidal endocrinology in free-ranging bottlenose dolphins (*Tursiops truncatus*). Gen Comp Endocrinol. (2019) 281:164–72. 10.1016/j.ygcen.2019.06.006 31199925 PMC6990413

[137] Beal A Kiszka JJ Wells RS Eirin-Lopez JM The Bottlenose dolphin Epigenetic Aging Tool (BEAT): a molecular age estimation tool for small cetaceans. Front Mar Sci. (2019) 6:561 10.3389/fmars.2019.00561

[138] Horvath S. DNA methylation age of human tissues and cell types. Genome Biol. (2013) 14:3156. 10.1186/gb-2013-14-10-r115 24138928 PMC4015143

[139] Levine ME Hosgood HD Chen B Absher D Assimes T Horvath S. DNA methylation age of blood predicts future onset of lung cancer in the women's health initiative. Aging. (2015) 7:690–700. 10.18632/aging.100809 26411804 PMC4600626

[140] Perna L Zhang Y Mons U Holleczek B Saum KU Brenner H. Epigenetic age acceleration predicts cancer, cardiovascular, and all-cause mortality in a German case cohort. Clin Epigenet. (2016) 8:64. 10.1186/s13148-016-0228-z 27274774 PMC4891876

[141] Roetker NS Pankow JS Bressler J Morrison AC Boerwinkle E. Prospective study of epigenetic age acceleration and incidence of cardiovascular disease outcomes in the ARIC study (atherosclerosis risk in communities). Circ Genom Precis Med. (2018) 11:e001937. 10.1161/CIRCGEN.117.001937 29555670 PMC5863591

[142] Shorter KA Shao Y Ojeda L Barton K Rocho-Levine J Van Der Hoop J A day in the life of a dolphin: Using bio-logging tags for improved animal health and well-being. Mar Mamm Sci. (2017) 33:785–802. 10.1111/mms.12408

[143] Mcdonald BI Johnson M Madsen PT. Dive heart rate in harbour porpoises is influenced by exercise and expectations. J Experim Biol. (2018) 221:jeb168740. 10.1242/jeb.168740 29122951

[144] Sayigh LS Tyack PL Wells RS Scott MD Signature whistles of free-ranging bottlenose dolphins *Tursiops truncatus*: stability and mother-offspring comparisons. Behav Ecol Sociobiol. (1990) 26:247–60. 10.1007/BF00178318

[145] Jones B Zapetis M Samuelson MM Ridgway S Sounds produced by bottlenose dolphins (*Tursiops*): a review of the defining characteristics and acoustic criteria of the dolphin vocal repertoire. Bioacoustics. (2019) 18:1–42. 10.1080/09524622.2019.1613265

[146] Bossart G. Marine mammals as sentinel species for oceans and human health. Vet Path. (2011) 48:676–90. 10.1177/0300985810388525 21160025

[147] Hall AJ Gulland FM Hammond JA Schwacke LH Boyd I Bowen W Epidemiology, disease, and health assessment. In: Boyd IL Don Bowen W Iverson SJ editors. Marine Mammal Ecology and Conservation. New York, NY: Oxford University Press (2010). p. 144–64.

[148] Gulland FM Dierauf LA Whitman KL CRC Handbook of Marine Mammal Medicine. 3rd ed Boca Raton, FL: CRC Press (2018).

[149] Hall MA Alverson DL Metuzals KI By-catch: problems and solutions. Mar Poll Bull. (2000) 41:204–19. 10.1016/S0025-326X(00)00111-9

[150] Fire SE Wang Z Byrd M Whitehead HR Paternoster J Morton SL Co-occurrence of multiple classes of harmful algal toxins in bottlenose dolphins (*Tursiops truncatus*) stranding during an unusual mortality event in Texas, USA. Harmful Algae. (2011) 10:330–6. 10.1016/j.hal.2010.12.001

[151] Mchugh KA Allen JB Barleycorn AA Wells RS Severe *Karenia brevis* red tides influence juvenile bottlenose dolphin (*Tursiops truncatus*) behavior in Sarasota Bay, Florida. Mar Mamm Sci. (2011) 27:622–43. 10.1111/j.1748-7692.2010.00428.x

[152] Weisberg RH Liu Y Lembke C Hu C Hubbard K Garrett M The coastal ocean circulation influence on the 2018 West Florida Shelf *K. brevis* red tide bloom. J Geophys Res Oceans. (2019) 124:2501–12. 10.1029/2018JC014887

[153] Zohdi E Abbaspour M Harmful algal blooms (red tide): a review of causes, impacts and approaches to monitoring and prediction. Int J Environ Sci Tech. (2019) 16:1789–806. 10.1007/s13762-018-2108-x

[154] Ortiz RM Worthy GA. Effects of capture on adrenal steroid and vasopressin concentrations in free-ranging bottlenose dolphins (*Tursiops truncatus*). Comp Biochem Phys A Phys. (2000) 125:317–24. 10.1016/S1095-6433(00)00158-6 10794960

[155] Norman SA Hobbs RC Foster J Schroeder JP Townsend FI A review of animal and human health concerns during capture-release, handling and tagging of odontocetes. J Cetacean Res Manag. (2004) 6:53–62. Available online at: https://pdfs.semanticscholar.org/e0dc/57f496a9434514ea187083bf672c7d10eeeb.pdf

[156] Mancia A Warr GW Chapman RW. A transcriptomic analysis of the stress induced by capture–release health assessment studies in wild dolphins (*Tursiops truncatus*). Mol Ecol. (2008) 17:2581–9. 10.1111/j.1365-294X.2008.03784.x 18466235

[157] Fair PA Schaefer AM Romano TA Bossart GD Lamb SV Reif JS. Stress response of wild bottlenose dolphins (*Tursiops truncatus*) during capture–release health assessment studies. Gen Comp Endocrinol. (2014) 206:203–12. 10.1016/j.ygcen.2014.07.002 25019655

[158] Hart LB Wells RS Kellar N Balmer BC Hohn AA Lamb SV. Adrenal hormones in common bottlenose dolphins (*Tursiops truncatus*): influential factors and reference intervals. PLoS ONE. (2015) 10:e0127432. 10.1371/journal.pone.0127432 25993341 PMC4436368

[159] Champagne CD Kellar NM Trego ML Brendan D Rudy B Wasser SK. Comprehensive endocrine response to acute stress in the bottlenose dolphin from serum, blubber, and feces. Gen Comp Endocrin. (2018) 266:178–93. 10.1016/j.ygcen.2018.05.015 29852162

[160] Wells RS Social structure and life history of bottlenose dolphins near Sarasota Bay, Florida: insights from four decades and five generations. In: Yamagiwa J Karczmarski L editors. Primates and Cetaceans. Tokyo: Springer (2014). p. 149–72.

[161] Broadhurst MK Bottlenose dolphins, *Tursiops truncatus*, removing by-catch from prawn-trawl codends during fishing in New South Wales, Australia. Mar Fish Rev. (1998) 60:9–14.

[162] Ingram SN Rogan E Identifying critical areas and habitat preferences of bottlenose dolphins *Tursiops truncatus*. Mar Ecol Prog Ser. (2002) 244:247–55. 10.3354/meps244247

[163] Díaz López B Interactions between Mediterranean bottlenose dolphins (*Tursiops truncatus*) and gillnets off Sardinia, Italy. J Mar Sci. (2006) 63:946–51. 10.1016/j.icesjms.2005.06.012

[164] Read AJ Drinker P Northridge S Bycatch of marine mammals in US and global fisheries. Conserv Biol. (2006) 20:163–9. 10.1111/j.1523-1739.2006.00338.x 16909669

[165] Brotons JM Grau AM Rendell L Estimating the impact of interactions between bottlenose dolphins and artisanal fisheries around the Balearic Islands. Mar Mamm Sci. (2008) 24:112–27. 10.1111/j.1748-7692.2007.00164.x

[166] Wells RS Allen JB Hofmann S Bassos-Hull K Fauquier DA Barros NB Consequences of injuries on survival and reproduction of common bottlenose dolphins (*Tursiops truncatus*) along the west coast of Florida. Mar Mamm Sci. (2008) 24:774–94. 10.1111/j.1748-7692.2008.00212.x

[167] Pulster EL Smalling KL Zolman E Schwacke L Maruya KA. Persistent organochlorine pollutants and toxaphene congener profiles in bottlenose dolphins (*Tursiops truncatus*) frequenting the Turtle/Brunswick River Estuary, Georgia, USA. Environ Contamin Toxic. (2009) 28:1390–9. 10.1897/08-240.1 19203137

[168] Wells RS Feeling the heat: potential climate change impacts on bottlenose dolphins. Whalewatcher. (2010) 39:12–7. Available online at: https://iucn-csg.org/wp-content/uploads/2010/03/Whalewatcher-Climate-Change-2010.pdf

[169] Bearzi G Agazzi S Bonizzoni S Costa M Azzellino A Dolphins in a bottle: abundance, residency patterns and conservation of bottlenose dolphins *Tursiops truncatus* in the semi-closed eutrophic Amvrakikos Gulf, Greece. Aquat Conserv Mar Freshw Ecosyst. (2008) 18:130–46. 10.1002/aqc.843

[170] Aliaga-Rossel E Conservation of the river dolphin (*Inia boliviensis*) in Bolivia. In: Garcia M Shostell J editors. Biology, Evolution and Conservation of the River Dolphins in South America and Asia: Unknown Dolphins in Danger. New York, NY: Nova Science Publishers (2010). p. 55–70.

[171] Braulik GT Noureen U Arshad M Reeves RR Review of status, threats, and conservation management options for the endangered Indus River blind dolphin. Biol Conserv. (2015) 192:30–41. 10.1016/j.biocon.2015.09.008

[172] Khan MS Factors affecting the survival of Indus River dolphin and species tolerance towards anthropogenic pressures. Mar Freshwat Res. (2017) 68:1245–50. 10.1071/MF16001

[173] Centelleghe C Da Dalt L Marsili L Zanetti R Fernandez A Arbelo M. Insights into dolphins' immunology: immuno-phenotypic study on Mediterranean and Atlantic stranded cetaceans. Front Immunol. (2019) 10:888. 10.3389/fimmu.2019.00888 31110505 PMC6499212

[174] Turvey ST Pitman RL Taylor BL Barlow J Akamatsu T Barrett LA. First human-caused extinction of a cetacean species? Biol Lett. (2007) 3:537–40. 10.1098/rsbl.2007.0292 17686754 PMC2391192

[175] Rojas-Bracho L Gulland FMD Smith CR Taylor B Wells RS Thomas PO A field effort to capture critically endangered vaquitas *Phocoena sinus* for protection from entanglement in illegal gillnets. Endang Species Res. (2019) 38:11–27. 10.3354/esr00931

[176] Wilkinson D Worthy GAJ Marine mammal stranding networks. Conserv Manage Mar Mamm. (1999) 2:396–411.

[177] Watwood SL Tyack PL Wells RS Whistle sharing in paired male bottlenose dolphins, *Tursiops truncatus*. Behav Ecol Sociobiol. (2004) 55:531–43. 10.1007/s00265-003-0724-y

[178] Bejarano AC Wells RS Costa DP Development of a bioenergetic model for estimating energy requirements and prey biomass consumption of the bottlenose dolphin (*Tursiops truncatus*). Ecol Model. (2017) 356:162–72. 10.1016/j.ecolmodel.2017.05.001

[179] Pasamontes A Aksenov AA Schivo M Rowles T Smith CR Schwacke LH. Noninvasive respiratory metabolite analysis associated with clinical disease in cetaceans: a deepwater horizon oil spill study. Environ Sci Technol. (2017) 51:5737–46. 10.1021/acs.est.6b06482 28406294

[180] Hall AJ Wells RS Sweeney JC Townsend FI Balmer BC Hohn AA. Annual, seasonal and individual variation in hematology and clinical blood chemistry profiles in bottlenose dolphins (*Tursiops truncatus*) from Sarasota Bay, Florida. Comp Biochem and Phys Part A Phys. (2007) 148:266–77. 10.1016/j.cbpa.2007.04.017 17524692

[181] Houser DS Finneran JJ Ridgway SH Research with navy marine mammals benefits animal care, conservation and biology. Int J Comp Psych. (2010) 23:249–68. Available online at: https://escholarship.org/uc/item/3pm7v89g

